# The enzymatic mechanisms of lactylation and its role in gynecological diseases: a comprehensive review

**DOI:** 10.3389/fimmu.2026.1828022

**Published:** 2026-04-30

**Authors:** Min Zhang, Yuanqi Li, Jinjin Chen, Yunxia Xu, Weidong Zhao

**Affiliations:** 1 The First Clinical College, Anhui University of Chinese Medicine, Hefei, China; 2 The First Affiliated Hospital, Anhui University of Chinese Medicine, Hefei, China

**Keywords:** gynecological diseases, lactylation, metabolic reprogramming, post-translational modification, therapeutic target

## Abstract

Lysine lactylation (Kla) is a novel form of post-translational modification. It utilizes lactate as its core substrate. Through an enzymatic regulatory network, it mediates modifications of both histones and non-histone proteins. This modification is thought to regulate gene transcription, reprogramming cellular metabolism, and remodeling the immune microenvironment. Studies on lactylation in gynecological diseases have progressively advanced. Its dysregulation appears to be associated with the pathogenesis of multiple conditions, including gynecological cancers, endometriosis, premature ovarian failure, and adverse pregnancy outcomes. Building upon the molecular regulatory mechanisms and disease associations of lactylation modification, several potential therapeutic strategies have been developed. These include targeting the enzymatic system, intervening in metabolic pathways, and implementing combination therapies. However, a systematic review summarizing the mechanistic role of lactylation modification in the development and progression of gynecological diseases, as well as its potential therapeutic value, is currently lacking. In light of this, this article reviews the regulatory network and metabolic correlation mechanisms of lactylation modification. It outlines its functional characteristics and clinical significance across various gynecological diseases. Furthermore, it summarizes therapeutic strategies that target this modification. The aim is to provide a reference for deeper research and clinical translation in this field, thereby supporting the evolution of precision diagnosis and treatment systems for gynecological diseases.

## 1 Introduction

Lactylation is a novel post-translational modification (PTM) first reported by Zhang’s team in 2019. It involves the covalent attachment of a lactate-derived lactyl group to the ϵ-amino group of a protein lysine residue, forming an Nϵ-lactyllysine modification site (1). This discovery offers an alternative to the traditional view of lactate as primarily a metabolic waste product. It demonstrates that lactate can act as a substrate for histone modification and regulate gene transcription. This opens a new perspective for cross-talk between metabolism and epigenetics.

Epigenetic regulation serves as a central bridge connecting environmental factors and gene expression (2). PTMs are the key effectors in this process. By dynamically modifying histones and epigenetics-related proteins, PTMs integrate external signals with intracellular metabolic states. This enables precise control of protein function and gene expression (3, 4). Lactylation, as a new type of PTM, links cellular metabolic status with transcriptional regulation. It plays important role in various physiological and pathological processes, including tumor proliferation, immune escape, cell differentiation, and stress response (5–7). An important feature of lactylation is its close association with cellular metabolism. Lactate, produced through glycolysis, is used to form lactyl modifications. This process involves “writers,” “erasers,” and “readers.” Together, they regulate downstream gene expression and activate signaling pathways (8).

Recent studies have suggested that lactylation plays a key role in gynecological diseases. For example, in gynecologic malignancies, lactylation promotes tumor progression and drug resistance by driving metabolic reprogramming and reshaping the immune microenvironment (9–11); In endocrine disorders such as premature ovarian failure, lactylation is involved in disease progression by regulating pathways related to follicular development and hormone synthesis (12, 13). In pregnancy-related conditions such as recurrent pregnancy loss, lactylation affects trophoblast function, placental metabolism, and maternal-fetal immune balance, leading to adverse pregnancy outcomes (14, 15). Therefore, this study focuses on the core molecular regulatory mechanisms and metabolic networks of lactylation. We aim to systematically review its functional characteristics, clinical significance, and therapeutic potential in gynecological diseases. This review aims to provide a reference for basic research and clinical translation in this field, and may support the development of precision diagnosis and treatment systems for gynecological diseases.

## 2 Lactate production and transport

Lactic acid is a key intermediate in glycolytic metabolism within organisms. It was long misunderstood as merely a “metabolic waste product” associated with hypoxic conditions. Recent studies have confirmed that lactate can specifically modify both histones and non-histone proteins through lactyl-CoA-mediated enzymatic reactions or non-enzymatic reactions. These modifications, in turn, regulate gene transcription and protein function (16). Consequently, lactate serves multiple physiological roles as an energy metabolite, a signaling molecule, and a precursor for epigenetic regulation. It is involved in various processes such as cellular metabolism, immune regulation, and tissue repair (16, 17). Lactate is primarily produced via the glycolytic pathway. Glucose is converted to pyruvate through glycolysis. Pyruvate is then transformed into lactate under the catalysis of lactate dehydrogenase A (LDHA). Conversely, lactate dehydrogenase B (LDHB) can catalyze the reverse reaction, converting lactate back to pyruvate (18). Under pathological conditions like hypoxia and inflammation, cells activate aerobic glycolysis, known as the Warburg effect. This results in substantial lactate accumulation, providing an ample substrate for lactylation modification (19). Furthermore, pathways such as glutaminolysis and fatty acid metabolism can indirectly influence lactate production through metabolism alteration, thereby modulating lactylation levels. Glutamine can enter the Tricarboxylic acid (TCA) cycle via glutaminolysis. It is then converted to pyruvate through catalysis by malic enzyme and further transformed into lactate. This provides an important non-glucose source of lactate for cells (20). In adipocytes, fatty acid metabolism interacts with the glycolysis pathway. This interaction alters the efficiency of pyruvate-to-lactate conversion, thereby also participating in the regulation of lactate production (21).

The transmembrane transport of lactate relies on the monocarboxylate transporter (MCT) family, which comprises 14 transmembrane proteins encoded by the SLC16A genes (22). The expression levels of MCTs are closely associated with cell type and metabolic status. Tumor cells frequently overexpress MCT4 to maintain intracellular lactate homeostasis, thereby supporting continuous lactylation modification (23). In most high-lactate microenvironments or common cell types, MCT1 mainly mediates the influx of extracellular lactate, while MCT4 is primarily responsible for the efflux of intracellular lactate. Together, they coordinate the distribution of lactate between the intracellular and extracellular spaces (24). However, recent studies have shown that the transport function of MCT4 is cell type-dependent. For example, in glioblastoma stem cells, MCT4 primarily mediates lactate import. This finding challenges the traditional view that MCT4 functions only as a lactate efflux transporter (25). In addition, the chaperone protein CD147 can form stable complexes with MCT1 and MCT4, enhancing their transport efficiency. This indirectly regulates intracellular lactate concentration and the level of lactylation modification (26, 27). In terms of kinetic characteristics, the MCT family shows significant isoform differences in lactate affinity. MCT1 has a high affinity for lactate, with a Michaelis constant (Km) of 3–5 mM. MCT2 has an even higher affinity, with a Km as low as 0.7 mM. In contrast, previous studies reported MCT4 as a low-affinity transporter, with a Km of approximately 28 mM (28). However, recent precise measurements using Förster resonance energy transfer (FRET)-based sensors show that the actual affinity of MCT4 under physiological conditions is higher than previously estimated, with a Km of approximately 0.7–1.7 mM for lactate (29). This suggests that MCT4 can still efficiently mediate lactate transport in high-lactate microenvironments, with its specific functional direction depending on cell type and metabolic context.

**Figure 1:**
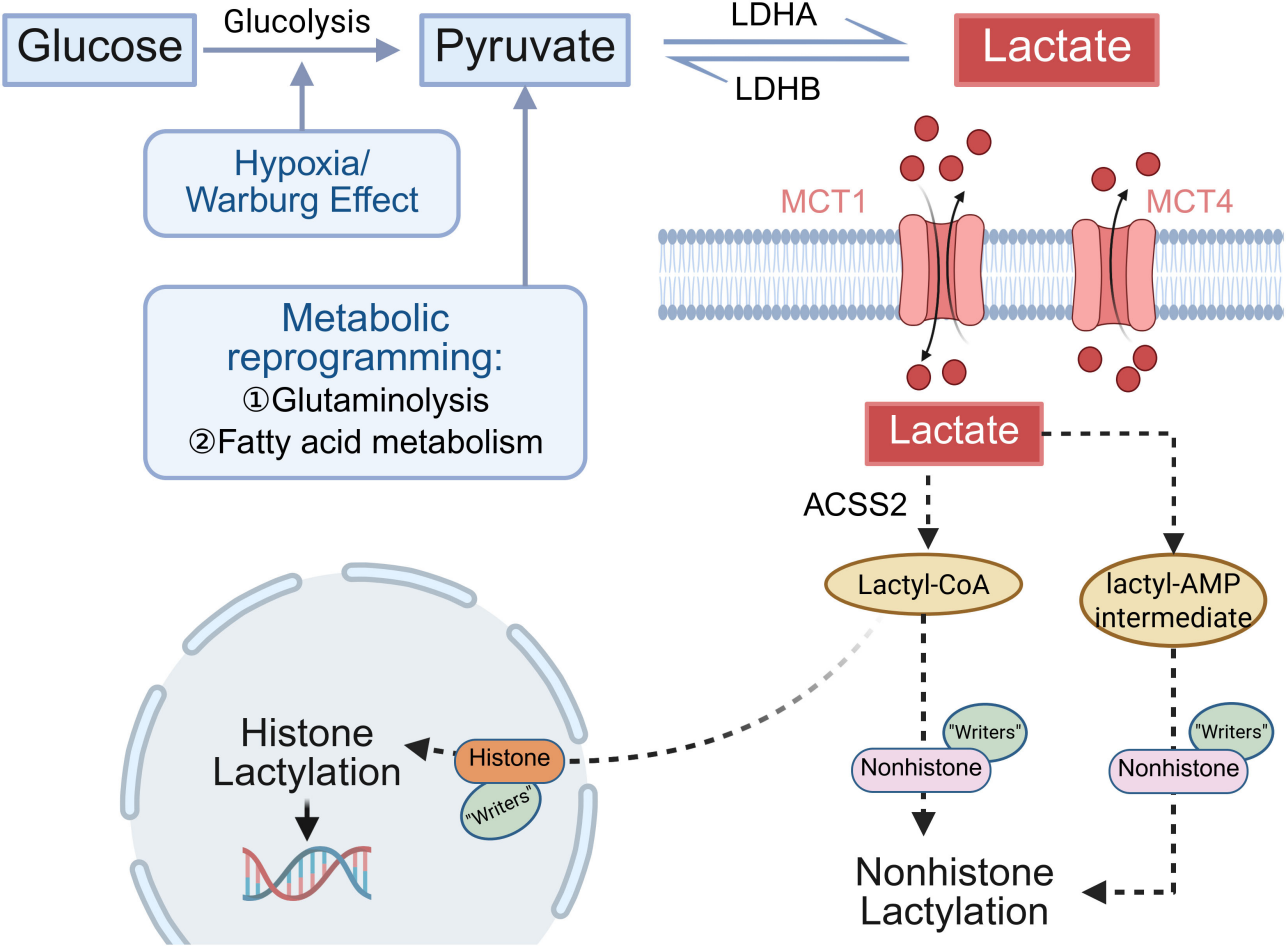
Metabolic pathway of lactate production, transmembrane transport, and its role as a substrate for lactylation modification. Glucose is converted to pyruvate via glycolysis. Pyruvate is reduced to lactate by LDHA, whereas LDHB catalyzes the reverse reaction. Hypoxia and the Warburg effect promote aerobic glycolysis, leading to lactate accumulation. In addition, glutaminolysis and fatty acid metabolism modulate lactate production through metabolic reprogramming. Lactate is transported across the membrane mainly by MCT1 and MCT4, the direction of transport depends on cell type. Intracellular lactate is converted to lactyl-CoA by ACSS2 via a lactyl-AMP intermediate. Lactyl-CoA serves as the acyl donor for lactylation of histones and nonhistone proteins, regulating gene transcription and protein function.

Intracellular lactate needs to be converted into lactyl-CoA to function as the acyl donor for lactylation. This conversion is primarily catalyzed by Acetyl-CoA synthetase 2 (ACSS2). Notably, lactate production, transport, and lactylation modification are not isolated processes, instead, they may form a functional closed loop through a “lactylation-aerobic glycolysis” positive feedback circuit. Specifically, lactylation promotes further lactate production by activating glycolytic gene expression and enhancing glycolytic enzyme activity. Conversely, the accumulated lactate subsequently elevates global lactylation levels through the transport and conversion mechanisms outlined above (30). This regulatory circuit exemplifies how lactate acts as both a metabolic intermediate and an epigenetic modulator. These processes are illustrated in **Figure 1**.

## 3 Dynamic regulatory mechanisms of lactylation modification

**Figure 2:**
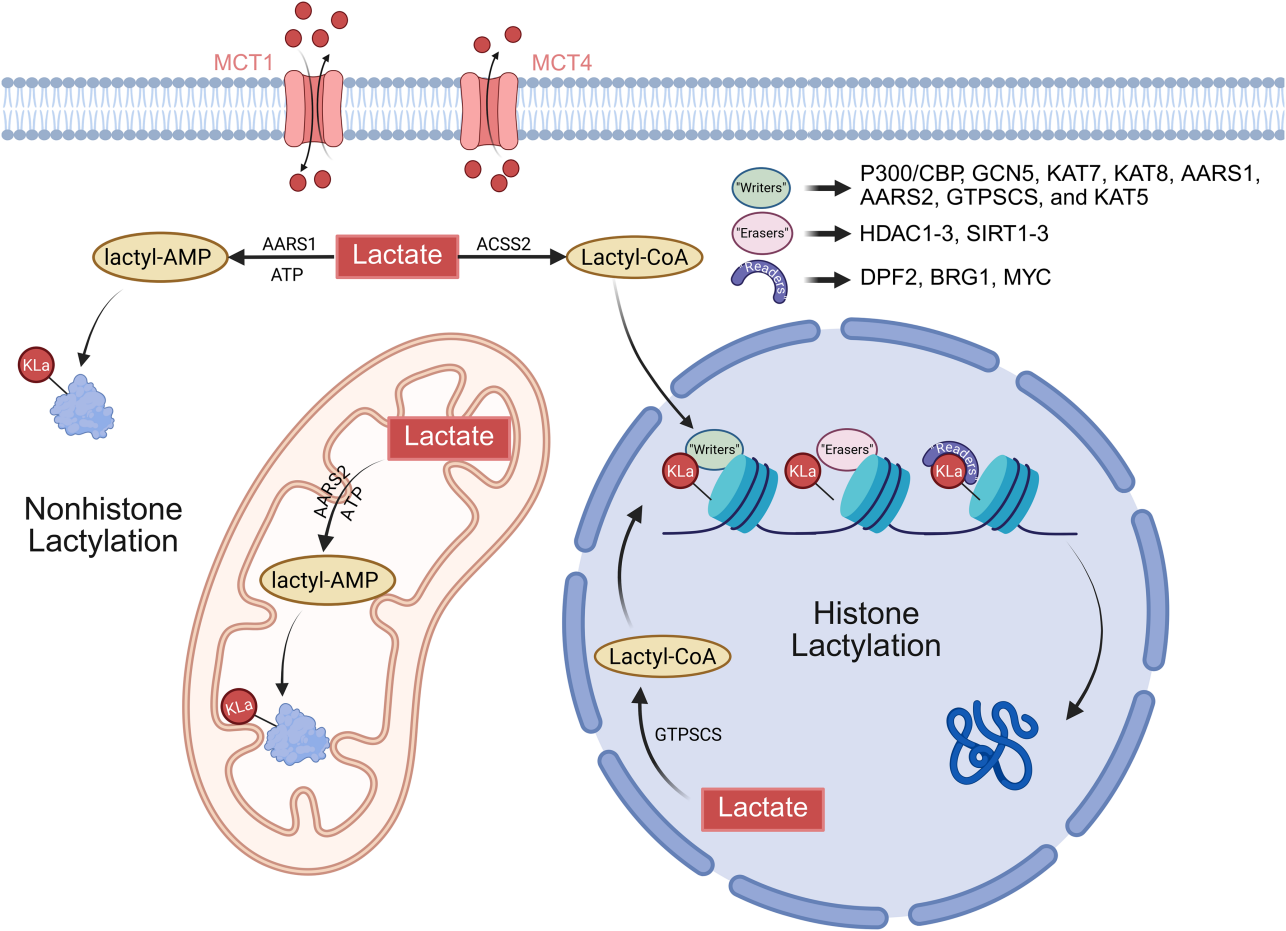
The dynamic regulatory network of lactylation modification. Lactate is converted to lactyl-CoA via ACSS2 (ATP-dependent) or GTPSCS (GTP-dependent) through a lactyl-AMP intermediate. AARS1/2 also generate lactyl-AMP. Lactyl-CoA serves as the acyl donor for histone and nonhistone lactylation catalyzed by writers including P300/CBP, GCN5, KAT7/8, and KAT5. Lactylation is removed by erasers HDAC1–3 and SIRT1–3. Lactate transport is mediated by MCT1 and MCT4. This network integrates lactate metabolism with writer–eraser dynamics to control protein lactylation.

The dynamic balance of lactylation modification is co-regulated by three components: “writers” (lactyltransferases), “erasers” (delactylases), and “readers” (lactylation readers). This network and key components are summarized in **Figure 2** and **Table 1**. Lactate, produced by cells through pathways such as glycolysis and glutaminolysis, is converted into lactyl-CoA under conditions of metabolic homeostasis or stress. This lactyl-CoA serves as the modification substrate and is recognized by the “writers” (1). These enzymes then catalyze the transfer of the lactyl group to lysine residues on both histones and non-histone proteins, thereby completing the modification. Subsequently, “readers” specifically recognize and bind to lactylation sites. This recognition facilitates the recruitment of downstream regulatory complexes. Thus, the lactylation signal may be transduced into specific biological outcomes, such as the regulation of gene expression, modulation of metabolic enzyme activity, or activation of signaling pathways. This signal transduction enables cells to either adapt to physiological demands or potentially contribute to pathological progression. Once cellular metabolism returns to a homeostatic state, the “erasers” are activated. These enzymes remove lactyl groups from proteins via hydrolysis of the amide bond, thereby terminating the lactylation signal (31). Simultaneously, the activity of the “writers” is inhibited, which can be attributed to substrate competition or a reduced availability of lactate. The coordinated interplay among these three components is closely associated with intracellular lactate concentration and the overall metabolic state. Furthermore, this feedback mechanism may help prevent aberrant hyper−lactylation. Ultimately, this system maintains homeostasis or contribute to metabolic reprogramming and functional remodeling in pathological contexts (32).

**Table 1:** Enzymes and reader proteins associated with lactylation modification.

Category	Name/acronym	Subcellular localization	Major substrates/target sites	Key features and functions	Ref.
Writers	p300/CBP	Nucleus	Histones: H3K18, H3K27, H4K12	Utilizes lactyl−CoA as acyl donor; exhibits 2.3−fold higher catalytic efficiency for lactylation versus acetylation; activated via AMPK−mediated phosphorylation under hypoxia, establishing a hypoxia-p300 activation-lactylation↑-glycolysis↑ regulatory axis.	(1, 33–36)
	GCN5 (KAT2A)	Nucleus	Histones: H3K18	Forms a functional complex with ACSS2, in which ACSS2 converts lactate to lactyl−CoA and GCN5 catalyzes the lactyl group transfer.	(34, 37, 38)
	KAT7 (HBO1)	Nucleus	Histones: H3K9	Identified as a writer enzyme that catalyzes lactylation at histone H3K9 (H3K9la) to regulate gene transcription.	(37)
	KAT8 (MOF)	Nucleus	Non-histones: eEF1A2	Catalyzes lactylation of translation initiation factor eEF1A2, upregulating protein translation rates.	(38)
	KAT5 (TIP60)	Nucleus	Non-histones: Vps34	Senses cellular lactate levels and catalyzes lactylation of the core autophagy protein Vps34, precisely regulating autophagy and lysosomal function.	(48, 49)
	AARS1	Cytoplasm	Non-histones	Lactyl−CoA−independent; directly uses L−lactate as substrate; functions as a lactate “sensor” with catalytic activity markedly enhanced by elevated lactate concentration; capable of nuclear translocation, promoting tumorigenesis.	(1, 41, 44)
	AARS2	Mitochondria	Non-histones: PDHA1 (K336), CPT2 (K457/K458) Histones: H3K18	Lactyl−CoA−independent; directly uses L−lactate as substrate; inactivates mitochondrial metabolic enzymes via lactylation, suppressing oxidative phosphorylation (OXPHOS); also mediates histone H3K18 lactylation, involved in processes such as ferroptosis.	(1, 42, 43)
	GTPSCS	Nucleus	Nuclear lactate	The first identified nuclear−localized lactyl−CoA synthetase; generates lactyl−CoA from L−lactate within the nucleus, supplying the precursor for localized histone lactylation.	(47)
Erasers	HDAC1-3	Nucleus	Histones: H3K18la, H4K12la, H4K5la	Class I histone deacetylases; confirmed as core “eraser” enzymes for histone lysine lactylation; isoforms exhibit differential recognition efficiency for lactylation sites (HDAC3 shows higher catalytic efficiency toward H3K9la and H4K12la; HDAC1/2 slightly prefer K(D−la) over K(L−la)); activity regulated by post−translational modifications.	(31, 50)
	HDAC6	Cytoplasm	Non-histones: α−tubulin (K40)	Exhibits lactate−concentration−dependent activity: promotes lactylation at >1 mM lactate, displays delactylation activity at <1 mM; regulates cytoskeletal function via metabolic signaling.	(50, 51)
	SIRT1	Nucleus/Cytoplasm	Non-histones: α-MHC (K1897)	NAD^+^−dependent deacylase; regulates lactylation of non−histone proteins (e.g., cardiac α−myosin heavy chain), contributing to the maintenance of cellular function.	(54)
	SIRT2	Cytoplasm	Non-histones: METTL16 (K229)	NAD^+^−dependent deacylase; specific delactylase for METTL16 at K229; modulates downstream signaling axes by regulating m^6^A modification.	(55, 56)
	SIRT3	Mitochondria	Histones: H3K9la	NAD^+^−dependent deacylase; primarily localized to mitochondria; exerts tumor−suppressive function by specifically removing lactylation at histone H3K9, thereby inhibiting pro−tumorigenic gene transcription.	(57, 58)
Readers	DPF2	Nucleus	Histones: H3K14la	Specifically recognizes and binds H3K14la via its PHD domain, thereby recruiting transcriptional co−activators to target gene promoters to drive gene transcription and tumorigenesis.	(59)
	BRG1	Nucleus	Histones: H3K18la	Core catalytic subunit of the SWI/SNF chromatin remodeling complex; recognizes histone H3K18la via its bromodomain to mediate chromatin remodeling, bridging glycolysis and lactylation modification.	(60)
	MYC	Nucleus	Histones: H4K12la	Oncogenic transcription factor; binds histone H4K12la via its basic helix−loop−helix (bHLH) domain, enhancing the transcriptional activity of its target genes.	(10)

### 3.1 Lactylation “writers”

Lactylation “writer” enzymes catalyze the transfer of lactyl groups onto lysine residues of substrate proteins. The known classes identified to date include members from the histone acetyltransferase (HAT) family and the Aminoacyl-tRNA synthetase (AARS) family. These two families differ in their catalytic mechanisms and substrate specificities.

Within the histone acetyltransferase (HAT) family, p300/CREB-binding protein (CBP) were the first enzymes identified as lactylation “writers.” They employ lactyl-CoA as an acyl donor to catalyze lactylation at specific histone residues, including H3K18, H3K27, and H4K12. This histone lactylation facilitates transcriptional activation by modulating chromatin architecture (33). The catalytic activity of these enzymes is coupled to the lactate metabolic pathway, with the cellular efficiency of lactyl-CoA synthesis influencing their capacity to install the modification (34). Notably, p300 catalyzes histone lactylation with an efficiency 2.3 times higher than that of acetylation, owing to the specific binding of lactoyl-CoA to its HAT domain (35). Under hypoxic conditions, AMPK-mediated phosphorylation activates p300. The activated p300 installs H3K18la modification, leading to the opening of promoter regions of key glycolytic genes, including GLUT1 and LDHA. This suggests a coherent regulatory axis where hypoxia induces p300 activation, which in turn enhances histone lactylation and ultimately may contribute to the transcriptional upregulation of glycolysis (1, 36).

General control non-depressible 5 (GCN5, also designated KAT2A) functions by forming a stable complex with ACSS2. In this functional unit, ACSS2 catalyzes the conversion of lactate to lactyl-CoA, providing the direct substrate. GCN5 then transfers the lactyl group specifically to the H3K18 site on histones. This GCN5-ACSS2 complex regulates genes involved in cellular metabolism (34). Furthermore, enzymes such as KAT7 and KAT8 have been reported to possess lactyltransferase activity. They can modify different histone sites or target non−histone protein substrates. Current research has identified that KAT7 primarily catalyzes lactylation at the histone H3K9 site (37), whereas KAT8 enhances protein translation rates by catalyzing the lactylation of the translation initiation factor eEF1A2 (38).

AARS1 and AARS2, members of the aminoacyl-tRNA synthetase (AARS) family, were identified as lactylation writer enzymes in 2024 by the team of Long Zhang et al (39). Enzymes within this family demonstrate pronounced substrate specificity. Their major targets are non−histone proteins, notably metabolic enzymes and signaling molecules (40). Through these modifications, they participate in important physiological processes such as metabolic homeostasis and the cellular stress response. Their catalytic mechanism operates independently of lactyl-CoA, utilizing L−lactate as the substrate. This is achieved through a dual−substrate recognition mechanism, whereby the enzyme concurrently binds L−lactate and the specific lysine residue on the target non−histone protein. This simultaneous binding facilitates the direct lactylation of the lysine residue (1).

As a cytoplasm-localized lactylation writer enzyme, AARS1 directly binds L−lactate. It then employs an ATP−dependent reaction to generate a lactyl−AMP intermediate. This activated intermediate subsequently facilitates the transfer of the lactyl group onto specific lysine residues of cytoplasmic target proteins, executing precise lactylation modifications (41). AARS2 is enriched in the mitochondrial matrix by its intrinsic targeting sequence. Like AARS1, it utilizes L−lactate as a substrate via an ATP−dependent catalytic mechanism. Specifically, AARS2 catalyzes lactylation at K336 of pyruvate dehydrogenase E1 alpha 1 (PDHA1) and at K457/K458 of Carnitine palmitoyltransferase 2 (CPT2) within the matrix. These modifications disrupt PDHA1 complex integrity and inhibit CPT2 activity, leading to reduced acetyl−CoA production. This suppression of acetyl−CoA flux consequently impedes oxidative phosphorylation (OXPHOS), shifting cellular metabolism from OXPHOS toward glycolysis. Ultimately, this metabolism alteration supports cellular adaptation to hypoxic conditions (42). On the other hand, AARS2 also mediates histone H3K18 lactylation, activates transcription of downstream genes including ACSL4 through chromatin remodeling, and thereby promotes ferroptosis during intestinal ischemia-reperfusion injury (43). Therefore, this distinct subcellular localization allows AARS1 and AARS2 to perform specialized functional roles. Each enzyme modifies substrates within its respective compartment—the cytoplasm for AARS1 and the mitochondria for AARS2—potentially contributing to spatial regulation of lactylation. Notably, the lactylation catalytic activity of the AARS family is regulated by intracellular lactate concentration. When intracellular lactate concentration increases to physiologically or pathologically relevant levels, the conserved lactate-binding domain in these enzymes undergoes a conformational change. This change enhances the domain’s binding affinity for lactate, leading to a significant increase in catalytic efficiency. For instance, *in vitro* treatment with 25 mM lactate can induce nuclear translocation of AARS1 and promote global lactylation (44); Conversely, the lactate concentration in tumors can reach approximately 40 mM (45), which enhances the catalytic efficiency of these enzymes. This increased efficiency mediates target protein lactylation and subsequent downstream pathway regulation (41, 46). This dose−responsive regulation establishes AARS enzymes as precise cellular “sensors” of metabolic status.

Furthermore, GTP−specific succinyl−CoA synthetase (GTPSCS) has been identified as the first nuclear−localized enzyme capable of synthesizing lactyl−CoA. It utilizes L−lactate as a substrate to catalyze the intranuclear production of lactyl−CoA, which in turn modulates histone lactylation. Notably, this regulatory function operates independently of canonical lactate metabolic pathways, including the mitochondrial TCA cycle, and does not interfere with other histone acyl modifications such as succinylation. Thus, GTPSCS represents a potential molecular mechanism for the compartmentalized regulation of lactylation specifically within the nucleus (47). KAT5 (also designated TIP60) has been further characterized as a pivotal “writer” enzyme that senses intracellular lactate levels. It catalyzes the lactylation of Vps34, a core autophagy−initiating protein. Moreover, KAT5 can target and modify inflammation−associated proteins. Through these modifications, it orchestrates precise regulation of both autophagy and lysosomal function (48, 49). The identification of these novel enzymes illustrates the expanding diversity and distinct functional specialization within the lactylation “writer” enzyme family.

### 3.2 Lactylation “erasers”

Lactylation modification is reversible. This reversibility is mediated by “eraser” enzymes, which remove lactyl groups from lysine residues. By doing so, they terminate the signaling effects initiated by lactylation and help maintain the dynamic equilibrium of the modification. The known eraser enzymes belong to two families: the histone deacetylases (HDACs) and the silent information regulators (SIRTs). Their regulatory activity exhibits both site specificity and a dependence on specific cofactors.

Class I histone deacetylases (HDAC1−3) function as “eraser” enzymes for histone Kla. They specifically recognize and remove lactyl groups from histone sites such as H3K18la and H4K12la (31). The delactylation activity of HDACs depends on specific interactions between certain residues within their catalytic domains and the substrate. Recognition of lactylation sites varies among HDAC isoforms. For instance, HDAC1, HDAC2, and HDAC3 all target the H4K5la site. Notably, HDAC3 demonstrates higher catalytic efficiency toward sites including H3K9la and H4K12la. It also exhibits hydrolytic activity against substrates modified by either L−lactate or D−lactate. In comparison, HDAC1 and HDAC2 show a slight preference for K(D−la) over K(L−la) modifications (31, 50). Research on Class II HDACs in relation to lactylation is still in its nascent phase. To date, HDAC6 has been primarily linked to this process. HDAC6 catalyzes lactylation specifically at the K40 residue of α−tubulin, thereby regulating cytoskeletal dynamics via metabolic signaling. Notably, its catalytic function exhibits a lactate−concentration−dependent switch: at intracellular lactate levels exceeding 1 mM, it promotes lactylation, while below 1 mM, it displays delactylation activity. This bifunctional regulation is dependent on the intrinsic activity of its deacetylase domain (50, 51). In addition, the activity of HDACs is also regulated by post−translational modifications. Evidence indicates that ubiquitination can promote the degradation of HDAC3. This degradation indirectly elevates the overall cellular level of lactylation (52).

Silent information regulators 1−3 (SIRT1−3) belong to the family of Nicotinamide adenine dinucleotide (NAD^+^)−dependent deacylases. Their catalytic mechanism relies on NAD^+^ as a coenzyme to participate in the acyl−transfer reaction (53). SIRT1 catalyzes the delactylation of both histone and non−histone protein substrates, contributing to the maintenance of metabolic homeostasis. For instance, in a heart failure model, SIRT1 modulates lactylation at the α−MHC K1897 site, which is essential for sustaining normal cardiomyocyte function (54). SIRT2 is primarily localized in the cytoplasm and preferentially modifies lactylation sites on cytoskeleton−associated proteins (55). Moreover, it serves as the specific delactylase for METTL16 at the K229 site. By regulating the METTL16/m^6^A/FDX1 axis, SIRT2 negatively regulates copper−dependent cuproptosis. Consequently, it may acts as a key inhibitor of cuproptosis in gastric cancer cells (56). SIRT3 is predominantly localized to mitochondria and modulates the activity of mitochondrial metabolic enzymes via delactylation modifications (57). In esophageal squamous cell carcinoma, SIRT3 has been associated with a tumor−suppressive role by specifically “erasing” lactylation at the histone H3K9 site. This enzymatic activity may leads to the repression of pro-tumorigenic gene transcription (58).

### 3.3 Lactylation “reader” proteins

Lactylation “reader” proteins recognize and bind to lactylated lysine residues via structural domains. This interaction mediates the activation of downstream signaling pathways or the regulation of gene transcription. The specificity of recognition is governed by the amino acid sequence surrounding the modification site and the local protein conformation. Identified reader proteins to date include Double PHD Fingers 2 (DPF2) and Brahma−Related Gene 1 (BRG1).

DPF2 functions as a reader protein for histone H3K14la. It engages H3K14la through its plant homeodomain (PHD) finger domain. Upon binding, DPF2 recruits transcriptional co−activators to the promoters of target genes, thereby promoting their expression. Studies reveal that the recognition specificity of DPF2 depends on hydrophobic amino acid residues flanking the lactylation site; mutating these residues impairs its binding affinity (59).

BRG1, a subunit of chromatin−remodeling complexes, recognizes histone H3K18la via its bromodomain to mediate chromatin remodeling. This function establishes a link between glycolysis and lactylation−driven epigenetic regulation (60). Furthermore, certain transcription factors and scaffold proteins have been suggested to possess the potential to recognize lactylation. For example, the oncogenic transcription factor MYC has been shown to bind to histone H4K12la through its basic helix−loop−helix (bHLH) domain, which may enhance the transcriptional activity of its target genes (10). Collectively, these reader proteins enable lactylation modifications to orchestrate gene expression networks by modulating processes such as transcription factor recruitment and chromatin remodeling. . .

## 4 Functional characteristics and clinical significance of lactylation modification in gynecological diseases

### 4.1 Gynecological malignancies

#### 4.1.1 Ovarian cancer

According to epidemiological surveys, the global age-standardized incidence rate of ovarian cancer is 6.6 per 100,000, with a mortality rate of 4.2 per 100,000. It has the highest mortality rate among gynecological malignancies, characterized by a high fatality rate and a low rate of early diagnosis (61, 62). Lactylation modification drives its progression, chemotherapy resistance, and immune evasion. For example, lactylation at the Phosphofructokinase, platelet type (PFKP) K392 site downregulates Phosphatase and tensin homolog (PTEN) expression, leading to PI3K/AKT pathway activation and consequently enhancing glycolysis and proliferation in ovarian cancer cells (63); moreover, lactate induces M2 polarization of macrophages via the surface receptor Gpr132, which subsequently activates CCL18 expression through H3K18la modification, thereby promoting ovarian cancer cell migration and metastasis (64); Single−cell RNA sequencing (scRNA−seq) further suggests that the Trefoil Factor 3 (TFF3+) epithelial cell subpopulation within ovarian cancer exhibits the highest lactylation levels, associated with advanced tumor progression (65).

Regarding chemotherapy resistance, GCN5−mediated H3K9la enrichment at the promoter regions of DNA repair genes (e.g., RAD51 and BRCA2) may enhances homologous recombination repair capacity in platinum−resistant ovarian cancer cells (66); in niraparib−resistant cells, accumulated lactate induces H4K12la modification, which activates the super−enhancer Nira−SE to drive RAD23A expression, thereby reinforcing DNA damage repair (10); Under low−glucose conditions, ACAT1−mediated ME2 acetylation promotes lactate production via glutaminolysis. The resulting lactate then modulates DNA repair proteins through lactylation, contributing to platinum−based drug resistance (67); Additionally, aberrant lactylation mediated by ALDH1A1 and S100A4 drives chemoresistance in ovarian cancer by regulating metabolic reprogramming (68).

In terms of immune evasion, LDHB−mediated regulation of H3K18la at the Programmed cell death ligand 1(PD−L1) promoter may promote PD−L1 expression and inhibits T−cell cytotoxic activity, facilitating tumor immune escape (69).

From a clinical perspective, high H3K18la expression correlates with poor prognosis in epithelial ovarian cancer patients and serves as an independent prognostic biomarker (70); A machine−learning model based on lactylation−related genes (TMEM126B, PYGL, NDUFS6) may predict patient prognosis and immunotherapy response. Notably, NDUFS6, identified as an oncogene, may represents a promising therapeutic target, with its inhibitor D−lactose showing potential as a targeted agent for ovarian cancer treatment (65).

#### 4.1.2 Endometrial cancer

Global epidemiological data indicate an increasing incidence of endometrial cancer (71). Lactylation modification contributes to its metabolic changes and disease progression. At the mechanistic level, H3K18la promotes tumor advancement by activating USP39 transcription, stabilizing the glycolytic enzyme PGK1, and subsequently stimulating the PI3K/AKT/HIF−1α pathway, thereby enhancing glycolytic flux in endometrial cancer cells. Accordingly, the glycolytic inhibitor 2-deoxy-D-glucose(2−DG) suppresses tumor progression by lowering lactylation levels, suggesting that targeting the USP39−PGK1−PI3K/AKT axis may offer a potential therapeutic strategy for this malignancy (72). Additionally, lactylation at the PFKM K678 site facilitates tumor cell proliferation, invasion, and angiogenesis (73). In the exploration of therapies targeting lactylation, physical treatments have also shown significant potential in addition to traditional drug interventions. Studies have confirmed that cold atmospheric plasma can inhibit H3K18la modification and activate the p53 pathway by regulating the USP49/HDAC3/H3K18la/p53 signaling axis. This, in turn, induces ferroptosis in endometrial cancer cells, offering additional evidence for the use of physical approaches to target lactylation in cancer therapy (52).

From a translational perspective, a risk model based on lactylation−associated differentially expressed genes (PFKM, H3C1, and SIRT3) screened from the TCGA database predicts overall survival in endometrial cancer patients and correlates with tumor mutational burden (TMB) and immune cell infiltration (74). Furthermore, another lactylation-related risk model, constructed based on IGSF1, ZFHX4, and SCGB2A1, may also accurately predict the prognosis and treatment response of patients with uterine corpus endometrial carcinoma (75).

#### 4.1.3 Cervical cancer

Cervical cancer is among the most common gynecological malignancies in women worldwide (76). Its pathogenesis is largely driven by persistent infection with high−risk human papillomavirus (HR−HPV) (77). Lactylation modification plays a role in both the oncogenic transformation and the modulation of the tumor immune microenvironment.

Regarding carcinogenic mechanisms, the HPV16 E6 protein suppresses lactylation at Glucose-6-phosphate dehydrogenase (G6PD) K45, thereby activating the pentose phosphate pathway and promoting cervical cancer cell proliferation (78). Additionally, lactylation at the DCBLD1 K172 site stabilizes G6PD, further contributing to tumor progression (79). Metabolic reprogramming leads to the accumulation of H3K14la, which recruits DPF2 to activate the transcription of pro−oncogenic genes and facilitate tumorigenesis (59). Consequently, key binding residues of DPF2, such as D274, represent potential therapeutic targets. Disrupting the DPF2−H3K14la interaction may offer a novel strategy for cervical cancer treatment. Concerning the regulation of the tumor immune microenvironment, lactate secreted by cervical cancer cells can induce H3K18la modification in macrophages. This modification upregulates GPD2 expression and promotes M2 polarization, thereby fostering an immunosuppressive microenvironment (80). Therapeutically, HDAC inhibitors can enhance chemosensitivity in cervical cancer by modulating lactylation levels (81), offering an additional rationale for combination therapy.

### 4.2 Gynecological endocrine diseases

#### 4.2.1 Endometriosis

Endometriosis (EMs) is a prevalent condition responsible for chronic pelvic pain and infertility. Aberrant regulation of lactylation modification is associated with its pathogenesis and progression. Ectopic endometrial tissues from patients exhibit elevated levels of lactate, increased LDHA expression, and heightened H3K18la modification. Mechanistically, lactate may promote the proliferation and invasion of ectopic endometrial cells via H3K18la-mediated activation of HMGB1 transcription and subsequent stimulation of the AKT/c-Myc/Cyclin D1 pathway (82). Furthermore, H3K18la in these cells activates METTL3 transcription, leading to m^6^A−dependent stabilization of HIF1A mRNA, which may in turn upregulate HMOX1 expression and confer resistance to ferroptosis. The combination of the glycolytic inhibitor 2−DG and the ferroptosis inducer erastin synergistically suppresses ectopic lesion growth (83), providing a potential combined therapeutic strategy for clinical management.

#### 4.2.2 Premature ovarian insufficiency

Premature ovarian insufficiency (POI) represents a significant disorder in female reproductive endocrinology, characterized by abnormal follicular development linked to dysregulated lactylation. Patients with POI show elevated serum concentrations of lactate and free fatty acids. A gain−of−function mutation in AARS2 (R199C) enhances its lactyltransferase activity, catalyzing lactylation−mediated inactivation of CPT2 and PDHA1. This results in accumulated free fatty acids and impaired oxidative phosphorylation, ultimately driving the depletion of primordial follicles. The AARS2 inhibitor β−alanine attenuates lactylation, reverses excessive follicular activation, and improves reproductive outcomes in POI mouse models (12), suggesting a promising pharmacological target for therapeutic development.

#### 4.2.3 Polycystic ovary syndrome

Polycystic ovary syndrome (PCOS) is intertwined with metabolic dysfunction, with lactylation modification playing a contributory role in its pathology. Granulosa cells from PCOS patients display upregulated Pyruvate kinase M2 (PKM2) expression and enhanced nuclear accumulation. Hyperandrogenemia facilitates PKM2 nuclear translocation through the ERK1/2 pathway, thereby inducing H3K9la and H3K18la modifications. These modifications drive three−dimensional genomic remodeling and activate key androgen−biosynthesis genes, including CYP17A1 and CYP11A1. Inhibition of PKM2 nuclear accumulation by TEPP−46 reverses the PCOS phenotype in murine models, restoring normal estrous cyclicity and reducing testosterone levels (13), indicating a potential avenue for targeted intervention in PCOS.

### 4.3 Pregnancy−associated diseases

#### 4.3.1 Recurrent spontaneous abortion

Recurrent spontaneous abortion (RSA) is closely associated with trophoblast dysfunction. Aberrant regulation of lactylation may be one of its important contributing factors. Current evidence suggests that the regulation of RSA by lactylation is context-dependent. Both excessive lactylation at specific sites and lactylation deficiency at critical sites can converge on trophoblast dysfunction through distinct mechanisms, plausibly increasing the risk of RSA.

In patients with spontaneous abortion, elevated lactate levels in chorionic tissue induce lactylation at the K36 residue of the transcription factor JunB, accelerating its ubiquitin−dependent degradation and thereby inhibiting trophoblast proliferation, migration, and invasion (14). Furthermore, a high-lactate environment upregulates S100A11 expression and activates the p38 MAPK/TGF-β1/SMAD pathway, leading to suppressed trophoblast function. In the same study, immune infiltration analysis based on lactylation−related core genes (HNRNPU, PTMA, CALD1, S100A11) further confirmed immune imbalance at the maternal−fetal interface (84). Other studies have also shown that histone H3K18la deficiency may lead to embryo implantation failure (85). Therefore, both excessive lactylation and lactylation deficiency can promote the development and progression of RSA by inhibiting trophoblast function, interfering with decidualization, and disrupting immune balance.

#### 4.3.2 Preeclampsia

Preeclampsia (PE) is a serious complication of pregnancy. Placental ischemia and hypoxia, oxidative stress, metabolic disturbances, and trophoblast dysfunction are important pathological factors in the development and progression of PE (86). As a key mode of metabolic-epigenetic regulation, lactylation also plays an important role in placental dysfunction. Hypoxia in placental tissues of PE patients appears to drive lactate accumulation, which could enhance H3K18la modification, activate GADD45A transcription, and promote trophoblast senescence along with accelerated placental aging (15). Reduced HLA−F expression in PE placentas suppresses PKM2 transcription while increasing lactylation at the PKM2 K305 site, an effect that may collectively impair trophoblast glycolysis and proliferation (87). Additionally, oxidative stress downregulates lactylation at the HK2 K346 residue, inhibiting its catalytic activity and mitochondrial localization and could thereby aggravating trophoblast injury (88).

#### 4.3.3 Recurrent implantation failure

Recurrent implantation failure (RIF) is closely tied to compromised endometrial receptivity, with lactylation playing a regulatory role. Endometrial tissues from RIF patients exhibit markedly reduced LDHA expression and diminished lactate production. Lactate promotes H3K18la modification, which upregulates SLC7A11 expression, thereby driving Epithelial-mesenchymal transition (EMT) and enhancing migration of endometrial epithelial cells. This process improves endometrial receptivity and supports blastocyst implantation. The lactate–H3K18la–SLC7A11 axis thus emerges as a pivotal pathway governing embryo implantation, offering a fresh molecular target for diagnosing and treating RIF (89).

#### 4.3.4 Systemic lupus erythematosus−associated adverse pregnancy outcomes

**Figure 3:**
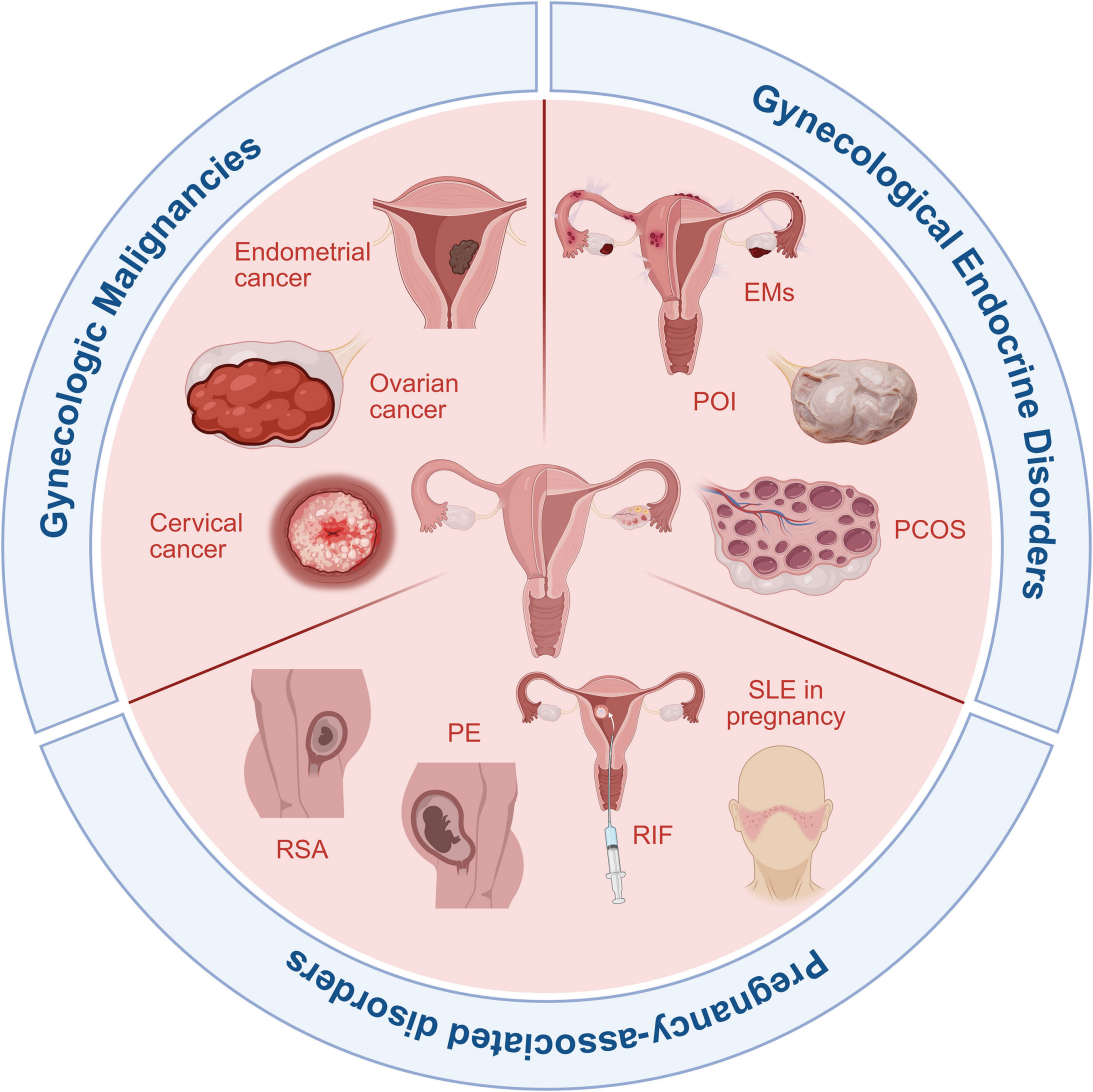
Overview of lactylation modification in gynecological diseases. Lactylation modification has been implicated in a broad spectrum of gynecological conditions. These include gynecological malignancies such as endometrial cancer, ovarian cancer, and cervical cancer; pregnancy-associated disorders including PE, preeclampsia; RSA, recurrent spontaneous abortion and SLE, systemic lupus erythematosus in pregnancy; as well as gynecological endocrine disorders such as Ems, endometriosis; POI, premature ovarian insufficiency, POI, polycystic ovary syndrome, and other endocrine disorders.

In SLE pregnancies, lactylationmediated trophoblast pyroptosis appears to constitute an important mechanism contributing to adverse outcomes. SLE pregnancies exhibit elevated neutrophil extracellular trap (NET) markers in both placental tissue and peripheral blood. NETs can activate trophoblast glycolysis, potentially leading to lactate production. This lactate may induces lactylation at the NLRP3 K166 residue, triggering NLRP3 inflammasome activation and subsequent pyroptosis. Inhibiting glycolysis or knocking down LDHA has been shown to reverse NET-induced pyroptosis. Interventions that suppress NET formation or target NLRP3 lactylation may therefore improve placental function, highlighting candidate therapeutic strategies for mitigating SLE related adverse pregnancy outcomes (90). The roles and clinical relevance of lactylation in gynecological diseases are summarized in **Figure 3** and **Table 2**.

**Table 2:** Functional and clinical correlations of lactylation modification in gynecological diseases.

Disease category	Disease name	Lactylation modification target/key gene	Primary functional effects and pathogenic mechanisms	Potential clinical applications/significance	Ref.
Gynecological Malignancies	Ovarian Cancer	PFKP (K392)	Downregulates PTEN, activates the PI3K/AKT pathway, and enhances glycolysis and proliferation.	Metabolic intervention may inhibit tumor progression.	(63)
		H3K18la	Induced by lactate in macrophages, activates CCL18 expression, and promotes cancer cell migration and metastasis.	Reveals a novel mechanism of immune−metabolic crosstalk within the tumor microenvironment.	(64)
		GCN5/H3K9la	Enriched at the promoters of DNA repair genes such as RAD51 and BRCA2 in platinum−resistant cells, enhancing homologous recombination repair.	Provides a potential target for overcoming platinum resistance (targeting GCN5 or lactylation).	(66)
		H4K12la	Activates super−enhancers in niraparib−resistant cells, driving RAD23A expression and enhancing DNA damage repair.	Explains a novel mechanism of PARPi resistance, suggesting a therapeutic strategy combining intervention in lactylation.	(10)
		LDHB/H3K18la	Regulates H3K18la at the PD−L1 promoter, promotes PD−L1 expression, and mediates immune evasion.	Provides a rationale for combining immune checkpoint inhibitors with lactate metabolism inhibitors.	(69)
		H3K18la	High expression is associated with poor prognosis in patients with epithelial ovarian cancer.	An independent prognostic marker that can be used for risk stratification.	(70)
		NDUFS6	A core oncogenic driver gene.	Its inhibitor (D−lactose) is a potential therapeutic agent.	(65)
	Endometrial Cancer	H3K18la	Activates USP39 transcription, stabilizes PGK1, and stimulates the PI3K/AKT/HIF−1α pathway, thereby enhancing glycolysis.	Targeting the USP39−PGK1 axis or using the glycolysis inhibitor 2−DG represents a potential therapeutic strategy.	(72)
		PFKM (K678)	Promotes tumor cell proliferation, invasion, and angiogenesis.	PFKM lactylation serves as a critical driver of tumor progression.	(73)
	Cervical Cancer	G6PD (K45)/DCBLD1 (K172)	HPV16 E6 inhibits G6PD lactylation to activate the PPP pathway, while DCBLD1 lactylation stabilizes G6PD; together they promote proliferation.	Reveals a novel metabolic−epigenetic mechanism underlying HPV−induced carcinogenesis and suggests that HDAC inhibitors may enhance chemosensitivity by modulating lactylation levels.	(78, 79)
		H3K14la/DPF2	Metabolic reprogramming leads to H3K14la accumulation, which recruits the reader protein DPF2 to activate pro−oncogenic gene expression.	DPF2 binding residues (e.g., D274) are potential drug targets.	(59)
		H3K18la/GPD2 (Macrophages)	Tumor−secreted lactate induces H3K18la in macrophages, upregulates GPD2, promotes M2 polarization, and fosters an immunosuppressive microenvironment.	Targeting lactate metabolism or the H3K18la/GPD2 axis reverses M2 macrophage polarization and remodels the anti-tumor immune microenvironment.	(80, 81)
Gynecological Endocrine Diseases	EMs	H3K18la/HMGB1	Lactate activates HMGB1 transcription via H3K18la, promoting cell proliferation and invasion through the AKT/c−Myc/Cyclin D1 pathway.	Adaptive growth mechanism of ectopic endometrial cells in the pelvic microenvironment	(82)
		H3K18la/METTL3-HIF1A	H3K18la activates METTL3 transcription, stabilizes HIF1A mRNA via m^6^A modification, upregulates HMOX1, and confers resistance to ferroptosis.	The combination of 2−DG and the ferroptosis inducer erastin synergistically inhibits lesion growth, offering a novel combinatorial therapeutic strategy.	(83)
	POI	AARS2/PDHA1, CPT2	A gain−of−function mutation in AARS2 catalyzes lactylation of mitochondrial PDHA1 and CPT2, leading to their inactivation, resulting in fatty acid accumulation and OXPHOS suppression, which drives primordial follicle depletion.	The AARS2 inhibitor β−alanine reverses the phenotype in mouse models and represents a potential therapeutic target.	(12)
	PCOS	PKM2 Nuclear Translocation/H3K9la, H3K18la	Hyperandrogenemia promotes PKM2 nuclear accumulation, induces histone lactylation, remodels the 3D genome, and activates androgen−synthesis genes.	The PKM2 nuclear accumulation inhibitor TEPP−46 reverses the PCOS phenotype in mouse models.	(13)
Pregnancy-Associated Diseases	RSA	JunB (K36)/S100A11	Elevated lactate induces JunB K36 lactylation in trophoblasts, accelerating its ubiquitin−mediated degradation. It also activates S100A11 expression, which suppresses trophoblast function via the p38 MAPK−TGF−β1−SMAD pathway.	Reveals a direct molecular mechanism by which lactate disrupts trophoblast function.	(14, 84)
	PE	H3K18la/GADD45A	Placental hypoxia drives lactate accumulation, which enhances H3K18la−mediated activation of GADD45A, inducing trophoblast senescence and accelerated placental aging.	Links placental metabolic dysregulation with cellular senescence.	(15)
		PKM2 (K305)/HLA-F	Reduced HLA−F expression inhibits PKM2 transcription and increases its lactylation at K305, leading to impaired glycolysis and proliferation in trophoblasts.	Elucidates a novel pathway through which HLA−F regulates trophoblast metabolism.	(87)
		HK2 (K346)	Oxidative stress downregulates HK2 lactylation at K346, suppressing its catalytic activity and mitochondrial localization, thereby exacerbating trophoblast injury.	Reveals an epigenetic bridge between oxidative stress and metabolic enzyme dysfunction.	(88)
	RIF	Lactate−H3K18la−SLC7A11 axis	Lactate−induced H3K18la upregulates SLC7A11, driving EMT and migration in endometrial epithelial cells to enhance receptivity.	Supplemental lactate or targeting this axis may represent a therapeutic strategy to improve endometrial receptivity.	(89)
	SLE-APO	NLRP3 (K166)	NETs activate trophoblast glycolysis, producing lactate that induces NLRP3 lactylation, inflammasome activation, and pyroptosis.	Inhibiting NETs or targeting NLRP3 lactylation may improve placental function, offering potential therapeutic targets for SLE pregnancies.	(90)

## 5 Conclusions and future perspectives

This review synthesizes the regulatory network and metabolic coupling mechanisms of lactylation, a recently identified post−translational modification. Starting from upstream metabolic processes—including lactate generation, transmembrane transport, and its conversion to lactyl−CoA—we dissect the dynamic enzymatic system comprising “writers” (e.g., p300/CBP, AARS family, GTPSCS), “erasers” (e.g., HDACs, SIRT1−3), and “reader” (e.g., DPF2, BRG1) proteins. We elucidate how lactylation establishes a “metabolism−epigenetics” crosstalk that connects cellular metabolic status with transcriptional outputs.

Moreover, this work delineates the functional landscape and clinical implications of lactylation across various gynecological pathologies. These encompass malignancies (e.g., ovarian, endometrial, and cervical cancers), endocrine disorders (e.g., endometriosis, premature ovarian insufficiency, polycystic ovary syndrome), and pregnancy−related complications (e.g., recurrent spontaneous abortion, preeclampsia, recurrent implantation failure).

Based on the molecular regulatory network of lactylation and its disease−associated characteristics, several potential therapeutic strategies have been developed. These primarily include two directions: targeting the lactylation enzymatic system and intervening in lactate metabolic pathways. Specifically, inhibiting “writer” enzymes, activating “eraser” enzymes, or blocking “reader” proteins can modulate lactylation levels. Additionally, glycolytic inhibitors, MCT inhibitors, and glutaminolysis inhibitors can reduce lactate production or regulate its transport by intervening in metabolic pathways. Lysine lactylation modification not only holds dual value as both a biomarker and a therapeutic target, offering a research perspective for the precise diagnosis and treatment of gynecological diseases, but more importantly, its reversible nature provides a critical theoretical foundation and practical rationale for developing new therapeutic strategies through intervention in its specific regulatory pathways.

Consequently, future research should prioritize the development of highly specific therapeutic agents targeting lactylation. These efforts need to be integrated with advanced analytical tools—such as single−cell sequencing and spatial metabolomics—to systematically map the cell−type specificity and spatiotemporal dynamics of lactylation modifications. Subsequently, rigorously designed multicenter clinical trials are essential to validate the safety and efficacy of such lactylation−targeted interventions. This translational pathway may accelerate the bench−to−bedside transition of lactylation research, potentially offering advances for precision management of gynecological diseases and supporting progress in gynecological medicine. .

## Glossary

2-DG: 2-deoxy-D-glucose
AARS: Aminoacyl-tRNA synthetase
ACSS2: Acetyl-CoA synthetase 2
AKT: Protein kinase B
BRG1: Brahma-related gene 1
CBP: CREB-binding protein
CPT2: Carnitine palmitoyltransferase 2
DPF2: Double PHD fingers 2
EMT: Epithelial-mesenchymal transition
EMs: Endometriosis
FRET: Förster resonance energy transfer
G6PD: Glucose-6-phosphate dehydrogenase
GCN5 (KAT2A): General control non-depressible 5
GTPSCS: GTP-specific succinyl-CoA synthetase
HAT: Histone acetyltransferase
HDAC: Histone deacetylase
HIF-1α: Hypoxia-inducible factor 1 alpha
Kla: Lysine lactylation
LDHA: Lactate dehydrogenase A
LDHB: Lactate dehydrogenase B
MCT: Monocarboxylate transporter
NAD⁺: Nicotinamide adenine dinucleotide
NET: Neutrophil extracellular trap
OXPHOS: Oxidative phosphorylation
PCOS: Polycystic ovary syndrome
PDHA1: Pyruvate dehydrogenase E1 alpha 1
PD-L1: Programmed cell death ligand 1
PE: Preeclampsia
PFKP: Phosphofructokinase, platelet type
PI3K: Phosphoinositide 3-kinase
PKM2: Pyruvate kinase M2
POI: Premature ovarian insufficiency
PPP: Pentose phosphate pathway
PTEN: Phosphatase and tensin homolog
PTM: Post-translational modification
RIF: Recurrent implantation failure
RSA: Recurrent spontaneous abortion
scRNA-seq: Single-cell RNA sequencing
SIRT: Silent information regulator
SLE: Systemic lupus erythematosus
TCA cycle: Tricarboxylic acid cycle
TFF3: Trefoil Factor 3
TMB: Tumor mutational burden.
